# Who Agrees to Review a Manuscript in *Ecology and Evolution* ? It Depends on Who Asks

**DOI:** 10.1002/ece3.74214

**Published:** 2026-09-04

**Authors:** Amy Yanagitsuru, Ivan C. C. Provinciato, Arley F. Muth, Jenny Ouyang

**Affiliations:** ^1^ Department of Biology University of Nevada Reno Nevada USA; ^2^ Department of Biology and Earth Systems Sciences Wilkes University Wilkes‐Barre Pennsylvania USA; ^3^ John Wiley & Sons Ltd. Hoboken New Jersey USA

**Keywords:** acceptance, editor, gender, gender equity, peer review, workload

## Abstract

Gender inequality in publishing is a well‐documented phenomenon especially for STEM fields. Thus far, however, the focus has been on authorship itself rather than the peer review process. Using reviewer responses from the past 5 years, we asked whether there is a gender bias in accepting to review a manuscript for articles submitted to *Ecology and Evolution*. Although men were asked twice as much as women to review a manuscript, we found no gender difference in agreeing to review a manuscript. However, we found that the gender of the editor asking does make a difference. To illustrate, men were more likely to agree to review a manuscript when asked by someone they likely perceived as a male editor than a female editor, whereas female reviewers were equally likely to agree regardless of their perception of the editor's gender. We urge further examination of these results to disentangle the specific underlying causes, as well as extensions into other fields and soliciting requests in academia.

## Graphical abstract

Our analysis reveals three key findings. First, men were invited to review manuscripts approximately twice as often as women, highlighting an imbalance in reviewer solicitation. Second, we found no overall difference between men and women in their likelihood of accepting review invitations, indicating that disparities in participation stem from invitation practices rather than willingness to review. Third, and most notably, we found that editor gender influences reviewer agreement rates: men were significantly more likely to accept review invitations when the request came from someone they likely perceived as a male editor, whereas women were equally likely to accept invitations regardless of editor gender. To our knowledge, this is the first study to document the influence of editor gender on reviewer participation in the peer‐review process.

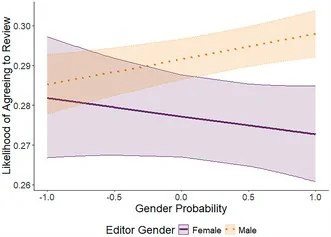

## 1 Introduction

By 2020, women were more likely than men to complete higher education and obtain university degrees worldwide (Encinas‐Martín and Cherian 2023). Nevertheless, gender inequality in academia persists. One of the most persistent manifestations of this imbalance is the so‐called “productivity puzzle,” which refers to the consistent finding that men publish more scientific papers over their careers than women (West et al. 2013; McDonald et al. 2020). The pieces to complete this puzzle are many, and several factors contribute to this disparity. For example, it is well documented that women remain underrepresented in graduate programs within Science, Technology, Engineering, and Mathematics (STEM) (Encinas‐Martín and Cherian 2023), contributing to their lower representation in senior academic positions such as full professorships (Larivière et al. 2013). Men, on average, have longer academic careers and are less likely to leave academia (Huang et al. 2020), while women often receive less recognition for their collaborative work or are under‐credited in authorship (West et al. 2013; Ross et al. 2022).

In addition, women frequently experience a disproportionate burden of balancing professional and personal responsibilities, which may lead them to prioritize flexibility and stability over competitiveness, ultimately constraining their research productivity (Encinas‐Martín and Cherian 2023). For instance, they are more often asked to undertake and are more inclined to accept service roles within academia—particularly routine service that is less visible and less prestigious—compared to men (O'Meara et al. 2017; Babcock et al. 2022). Women are less likely to hold high‐profile service positions, such as editorships or committee chair roles, and much of the service work they perform does not appear on their *curriculum vitae* (O'Meara et al. 2017). Consequently, while men have more time to dedicate to research and building collaborations, the disproportionate service burden placed on women further reduces the time they can invest in advancing their own careers (Miller and Roksa 2020).

To ensure fair and rigorous evaluation of research, the peer‐review process is indispensable—it upholds the integrity of scientific publishing and depends entirely on the voluntary participation of researchers, editors, and authors. In this process, authors submit manuscripts for evaluation, while editors invite qualified scientists from different career levels to assess the paper's quality and validity. Different studies have already identified several gender‐related biases in the peer‐review process, including lack of diversity in editorial boards (Fox et al. 2016, 2019), low proportion of female reviewers (Fox et al. 2019), and lower acceptance of review invitations by men when the editor has a female perceived name (Fox et al. 2016). In addition, while women invited to reviews are less likely to respond when compared to men (Fox et al. 2016), they are more likely to accept when they do respond (Fox et al. 2016, 2019; Schmaling and Blume 2017), often taking longer times to complete their reviews than men (Schmaling and Blume 2017). Furthermore, the peer‐review process for papers authored by women is often longer, with referees spending more time reviewing these manuscripts, requiring additional rounds of review, and taking longer to recommend acceptance (Alexander et al. 2023). Finally, during the first wave of the COVID‐19 pandemic, although an increase in female referees was observed—except in health and medicine journals, where this may have directly affected COVID‐19‐related research output—men submitted more manuscripts than women, further illustrating how the pandemic affected the careers of men and women in different ways (Squazzoni et al. 2021).

Gender disparities in academic recognition are well documented, such as the under‐acknowledgment of women's contributions (Lincoln et al. 2012; Llorens et al. 2022; Chalmers and Toribio‐flórez 2024). Studies on peer‐review bias toward nonbinary researchers are limited; however, existing research documents publishing‐related barriers that disproportionately affect nonbinary scientists (Armada‐Moreira et al. 2021; Nolan et al. 2025). Other biases also shape publication outcomes, including the lower acceptance rates of manuscripts from non‐English‐speaking countries (Primack et al. 2009).

Using a dataset from *Ecology and Evolution* editors and reviewers, we tested the simple null hypothesis that women and men are equally likely to agree to review a paper. As reviewers are anonymous (in the case of *Ecology and Evolution*), reviewers are not compensated financially or with recognition for their time. We note that some journals publish an annual report of reviewers who reviewed for the journal, but very rarely do these lists receive recognition for merit.

## 2 Methods

Data were sourced from *Ecology and Evolution* editorial records through the ScholarOne submission platform from year 2020 to 2025. We included the years 2020–2025 to be temporally relevant but still have enough data for analysis. Data included reviewer and editor name, the country the reviewer was employed in, and the reviewer's response to the review request. Once the relevant information had been extracted, all identifying reviewer and editor information was discarded. After filtering for reviewers whose names were abbreviated to one letter or contained typos (*n* = 7753), the dataset included 79,649 review requests sent to 40,780 unique reviewers from 222 unique editors. Responses included agreed, auto‐declined (no response), declined, no response, unavailable, or late response. These response categories were collated to include no responses and unavailable as a decline. Late responses were removed.

Genders were predicted using the commercial gender prediction service Genderize.io (“Genderize Documentation”) using reviewer first names and country of employment. We acknowledge that predictive algorithms have biased error rates: for example, female and Asian (especially Chinese) names are more frequently misclassified than male or non‐Asian names (Lockhart et al. 2023). Furthermore, because gender predictive algorithms classify gender on a binary, all nonbinary subjects are misclassified. Despite these known issues, Genderize.io is currently one of the most accurate predictive tools currently available (VanHelene et al. 2024) and with these caveats and known error rates, gender predictive algorithms can provide useful information.

Genderize.io outputs whether a first name is generally male or female in the provided country and the probability associated with the gender prediction. We predicted genders for 13,164 reviewer first names using name and country information, and on our first pass, Genderize reported 4661 of first names as unknown gender. To generate predictions for these remaining names, we removed country information and put these 4661 names through Genderize again, leaving only 655 names with unknown genders, approximately 5% of the total. The gender of approximately 30% of the names in our dataset were therefore predicted without country information.

In this study, reviewer gender is a latent variable—not directly observed, but with a known level of uncertainty provided by Genderize.io. Following the framework proposed by Prentice for managing cases where group identity is measured with error (Prentice 1982), we coded reviewer gender predicted from country and first name on a scale from −1 to 1 using the probability associated with gender prediction, where a value of −1 corresponds to a first name being certainly female and a value of 1 being certainly male. Although editor genders are known in our dataset, we treated editor gender the same as reviewer gender because potential reviewers do not have immediate information on the gender of the editor inviting them to review. Predicted editor gender rather than actual gender more accurately reflects how a potential reviewer may perceive an editor.

This research was deemed exempt by the University of Nevada Reno IRB (Project number: 2435640‐1).

### 2.1 Statistical Analysis

All analyses were conducted using R 4.5.1. All statistical models were binomial generalized linear mixed effects models with manuscript ID as a random effect unless otherwise specified. Model specifications are reported in Table S1.

### 2.2 Validation of Gender Predictions

We validated the predictions of reviewer gender from Genderize.io using the 222 editors in our dataset, who had known genders. Editor gender was predicted using Genderize.io without country information for a more conservative estimate of error rate, since the gender of approximately 30% of reviewer first names was predicted without country information. Using this validation dataset, we simply calculated the proportion of times Genderize.io's prediction was correct to get an error rate. We also evaluated whether the reported gender probability reflected predictive accuracy using a binomial generalized linear model.

## 3 Results

Validation of gender predictions from Genderize.io based on known‐gender editors revealed that Genderize.io predicted genders correctly 89.2% of the time. Genderize.io reported a higher gender probability when the editor's gender was predicted correctly (estimate = 9.63, SE = 1.84, *p* < 0.001), indicating that the gender probabilities accurately reflect uncertainty. There were more male reviewers than female reviewers in our dataset (51,078 male reviewers vs. 27,172 female reviewers).

**FIGURE 1:**
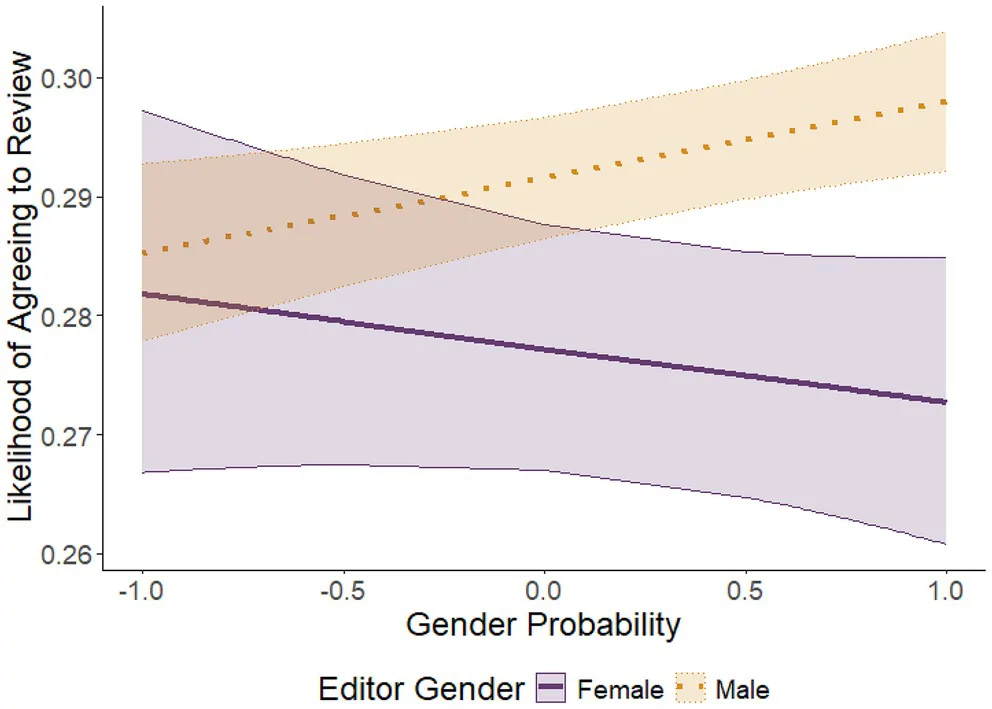
Male reviewers are more likely to agree to review when asked by an editor with a name that was predicted male. “Gender Probability” refers to the predicted gender of the reviewer, with female names < 0 and male names > 0. Editor gender is shaded in purple for female and orange and dotted line for male.

Although there was no difference in the likelihood of agreeing to review in men and women (*p* = 0.73), the perceived gender of the editor mattered, where male reviewers were slightly more likely to agree to review than female reviewers when asked by an editor with a male‐sounding name (estimate = 0.027, SE = 0.014, *p* = 0.049; Figure 1). For female reviewers, there was no effect of perceived editor gender (slope = 0.009, SE = 0.023, probability of agreeing = 28%). Male reviewers, however, had a 12.8% higher log‐odds of agreeing to review when asked by an editor with a male‐sounding name compared with a female‐sounding name (95% CI = 5.4%–22%). This corresponds to a probability of 27% of agreeing to review when asked by a female editor and a 30% probability of agreeing to review when asked by a male editor.

Men were slightly more likely to respond to the request to review than women (difference in log‐odds = 0.034, SE = 0.012, *p* = 0.007), with a response probability of 78.3% in women and 79.4% in men. There was no effect of perceived editor gender on the likelihood of responding. There was no gender difference in whether potential reviewers responded to the request to review on time.

## 4 Discussion

We tested whether men or women were more likely to accept to review a paper in *Ecology and Evolution* given that reviewing a paper, although an essential role for publishing, receives little to no recognition in academia. We found that there was no difference in acceptance to review based on gender (~30% for both sexes), but that the gender of the editor asking matters. Men were more likely to review a paper when asked by someone they perceive as a male than female editor, but women were equally likely to review regardless of the editor gender. This same pattern was previously reported in *Functional Ecology* (Fox et al. 2016), although it was not observed in journals such as *Journal of Animal Ecology*, *Journal of Applied Ecology*, *Journal of Ecology*, *Methods in Ecology and Evolution*, and *Evolution* (Fox et al. 2019). These studies also documented additional biases across all six journals, including the underrepresentation of women on editorial boards and among reviewers in *Ecology and Evolution* journals (Fox et al. 2016, 2019). Although female representation among editors (29% in 2015) and reviewers (27% in 2015) increased gradually over time, these numbers remain low. Importantly, our findings indicate that the same bias identified in *Functional Ecology* more than a decade ago persists currently in *Ecology and Evolution*, potentially influencing not only publication outcomes but also the career advancement of female researchers.

Several factors may contribute to this pattern. Women continue to be underrepresented in many STEM fields, which limits their visibility and, consequently, the likelihood of being invited as reviewers (Encinas‐Martín and Cherian 2023). In addition, persistent inequities in authorship and citation practices can reduce the recognition female scientists receive (West et al. 2013; Ross et al. 2022), making them less readily identified as experts. Furthermore, the structural and social barriers women face in academic environments, including fewer networking opportunities and the impacts of sexist climates, may further constrain their professional connections (O'Meara et al. 2017; Babcock et al. 2022), ultimately decreasing the probability of being asked to review scholarly work. Another possible explanation involves editors' professional networks (Helmer et al. 2017). A study using data from the *Frontiers* journal series showed that editors of both genders exhibit strong same‐gender preferences when selecting reviewers (Helmer et al. 2017), a phenomenon known as homophily (McPherson et al. 2001). Because same‐gender professional ties are generally more common, and because editorial boards remain disproportionately composed of men (Fox et al. 2016, 2019), this mechanism may also contribute to the patterns observed in our study. As editors are likely to draw upon their personal networks, cases in which editors cannot draw on their networks may result in them inviting a different gendered reviewer and making a poor fit selection, which would increase the chances that a reviewer rejects an invitation. These alternative explanations may not be mutually exclusive, and the mechanisms behind the phenomenon are difficult to disentangle.

Although the precise mechanism behind this pattern remains unclear, it may suggest that women editors face greater challenges in securing reviewers—particularly given that the reviewer pool is already male‐dominated. This dynamic could also imply that manuscripts handled by women editors take longer to move through the review process, but we lack the ability to disentangle these mechanisms. It is important to note that this pattern likely will not extend to journals that employ fully anonymous editorial systems (Fox et al. 2016). *Ecology and Evolution* displays editor names in reviewer invitations for several reasons, including promoting transparency to support reviewer recruitment and accommodating editors' preferences to be identified. However, this practice may benefit some editors more than others and could inadvertently introduce gender‐based biases. Possible solutions, such as omitting the editors' names could also imply in other problems/biases: editors would need to completely give up on recognition and visibility that they might gain from their editorial contributions (Fox et al. 2016). For the reviewers perspective, not knowing who is requesting the review could negatively impact reviewer recruitment (Fox et al. 2016). A possible compromise is using first name initials of editors' names for correspondence. Finally, this phenomenon could extend beyond the peer review process, that is, department chairs soliciting letters for tenure evaluations and further research beyond the peer‐review process could reveal previously unidentified biases.

There are some caveats associated with our study. First, genderize.io is not the perfect classification of gender due to misclassification of female, Asian, and non‐binary names (Lockhart et al. 2023). Gender is not self‐identified during the peer‐review process at *Ecology and Evolution*, and because gender could only be inferred using indirect methods, potential biases affecting nonbinary researchers could not be distinguished in this study. This limitation contributes to a persistent knowledge gap regarding how nonbinary researchers experience peer review, and it will be essential to re‐evaluate this issue when more accurate identity data becomes available. Regarding the manuscripts themselves, we did not analyze the gender of first or last authors, country of origin, or whether the submission was ultimately accepted. As other studies have shown, these are factors that also bias manuscript review and acceptance (Helmer et al. 2017; Fox et al. 2023). Future work examining these variables together is essential to identify additional biases that may be embedded in the peer‐review process. Moreover, our findings may not generalize across scientific disciplines, underscoring the need for similar analyses in other journals and fields. Our work shows that gender biases in the peer review process found over a decade ago in ecology journals still exist. We found that the gender of the editor matters for male reviewers and are interested in whether these patterns extend beyond *Ecology and Evolution*.

## Author Contributions

**Amy Yanagitsuru:** methodology (equal), writing – original draft (equal), writing – review and editing (equal). **Ivan C. C. Provinciato:** writing – original draft (equal), writing – review and editing (equal). **Arley F. Muth:** conceptualization (equal), investigation (lead). **Jenny Ouyang:** conceptualization (equal), methodology (equal), supervision (lead), writing – original draft (equal), writing – review and editing (equal).

## Funding

J.O. is funded by National Science Foundation (NSF) award IOS‐2141693.

## Conflicts of Interest

The authors declare no conflicts of interest.

## Supporting information


**Table S1:** Independent and dependent variables for binomial generalized linear mixed effects models. For all models manuscript ID was included as a random effect.

## Data Availability

Data and code available at https://doi.org/10.5061/dryad.mcvdnckf2.
