# Supplementary material for: Who Agrees to Review a Manuscript in Ecology and Evolution ? It Depends on Who Asks
Source: Ecol Evol. 2026 Sep 4;16(9):e74214. doi: 10.1002/ece3.74214 (PMC13545504; doi:10.1002/ece3.74214)
Supplement: Supplementary file 1 [file ECE3-16-e74214-s001.docx]

**Supplementary materials for:** Who agrees to review a manuscript in *Ecology and Evolution*? It depends on who asks

Amy Yanagitsuru^1,2^*, Ivan Provinciato^1^*, Arley F. Muth^3^, Jenny Ouyang^1^

*Co-first authors, authors contributed equally

^1^ Department of Biology, University of Nevada, 1664 N Virginia St, Reno, Reno, NV, USA

^2^ Department of Biology and Earth Systems Sciences, Wilkes University, 84 W South St, Wilkes-Barre, PA, USA

^3^ John Wiley & Sons Ltd, Hoboken, NJ, USA

Corresponding author: iprovinciato@unr.edu

| Outcome predicted | Independent variable(s) |
| --- | --- |
| Likelihood of agreeing to review | Reviewer gender probability x editor gender probability |
| Likelihood of men agreeing to review | Editor gender probability |
| Likelihood of women agreeing to review | Editor gender probability |
| Likelihood of responding to the request to review | Reviewer gender probability x editor gender probability |
| Likelihood of responding to the request to review on time | Reviewer gender probability x editor gender probability |

**Table S1:** Independent and dependent variables for binomial generalized linear mixed effects models. For all models manuscript ID was included as a random effect.
